# Glucagon Receptor Signaling and Lipid Metabolism

**DOI:** 10.3389/fphys.2019.00413

**Published:** 2019-04-24

**Authors:** Katrine D. Galsgaard, Jens Pedersen, Filip K. Knop, Jens J. Holst, Nicolai J. Wewer Albrechtsen

**Affiliations:** ^1^Department of Biomedical Sciences, Faculty of Health and Medical Sciences, University of Copenhagen, Copenhagen, Denmark; ^2^Novo Nordisk Foundation Center for Basic Metabolic Research, Faculty of Health and Medical Sciences, University of Copenhagen, Copenhagen, Denmark; ^3^Department of Cardiology, Nephrology and Endocrinology, Nordsjællands Hospital Hillerød, University of Copenhagen, Hillerød, Denmark; ^4^Clinical Metabolic Physiology, Steno Diabetes Center Copenhagen, Gentofte Hospital, Hellerup, Denmark; ^5^Department of Clinical Medicine, Faculty of Health and Medical Sciences, University of Copenhagen, Copenhagen, Denmark; ^6^Department of Clinical Biochemistry, Rigshospitalet, Copenhagen, Denmark; ^7^Novo Nordisk Foundation Center for Protein Research, Faculty of Health and Medical Sciences, University of Copenhagen, Copenhagen, Denmark

**Keywords:** glucagon, lipid, liver, adipose tissue, alpha cell

## Abstract

Glucagon is secreted from the pancreatic alpha cells upon hypoglycemia and stimulates hepatic glucose production. Type 2 diabetes is associated with dysregulated glucagon secretion, and increased glucagon concentrations contribute to the diabetic hyperglycemia. Antagonists of the glucagon receptor have been considered as glucose-lowering therapy in type 2 diabetes patients, but their clinical applicability has been questioned because of reports of therapy-induced increments in liver fat content and increased plasma concentrations of low-density lipoprotein. Conversely, in animal models, increased glucagon receptor signaling has been linked to improved lipid metabolism. Glucagon acts primarily on the liver and by regulating hepatic lipid metabolism glucagon may reduce hepatic lipid accumulation and decrease hepatic lipid secretion. Regarding whole-body lipid metabolism, it is controversial to what extent glucagon influences lipolysis in adipose tissue, particularly in humans. Glucagon receptor agonists combined with glucagon-like peptide 1 receptor agonists (dual agonists) improve dyslipidemia and reduce hepatic steatosis. Collectively, emerging data support an essential role of glucagon for lipid metabolism.

## Introduction

Glucagon is processed from its precursor, proglucagon, by prohormone convertase 2 and secreted from pancreatic alpha cells (Rouille et al., 1994). The role of glucagon in glucose metabolism has been intensively studied, and comprehensive reviews are found elsewhere (Jiang and Zhang, 2003; Ramnanan et al., 2011; Ahren, 2015; Holst et al., 2017a). In addition to regulating glucose metabolism, glucagon also seems important for minute-to-minute regulation of amino acid metabolism as part of the recently described liver-alpha cell axis (Solloway et al., 2015; Dean et al., 2017; Galsgaard et al., 2017; Holst et al., 2017b; Kim et al., 2017), in which amino acids stimulate glucagon secretion and glucagon in turn stimulates hepatic amino acid uptake and metabolism (ureagenesis) and, thus, circulating amino acid concentrations as well as increased hepatic NADH/NAD^+^ ratio. The actions of glucagon are mediated via the glucagon receptor, a seven transmembrane receptor coupled to G_αs_- and G_q_-proteins, which regulate adenylate cyclase (AC) and phospholipase C activities when activated (Wakelam et al., 1986; Jelinek et al., 1993; Aromataris et al., 2006). The glucagon receptor is primarily expressed in the liver, but it is also expressed in varying amounts in the central nervous system, kidneys, gastro-intestinal tract, heart (controversial), and pancreas (Svoboda et al., 1994).

Glucagon receptor expression has been reported in rat adipocytes (Svoboda et al., 1994; Hansen et al., 1995), where a lipolytic effect of glucagon may be of physiological relevance. As type 2 diabetic hyperglucagonaemia (Faerch et al., 2016) contributes to the hyperglycemic state of patients with type 2 diabetes (T2D) (Unger and Orci, 1975; Baron et al., 1987), inhibition of glucagon receptor signaling has been investigated as glucose-lowering therapy in T2D patients (Kazda et al., 2016; Kazierad et al., 2016, 2018; Vajda et al., 2017; Pettus et al., 2018). Interestingly, potential adverse effects of this therapeutic approach include increased low-density lipoprotein (LDL) plasma concentrations and increased hepatic fat accumulation (Guzman et al., 2017). Furthermore, hepatocyte studies have shown that glucagon stimulates beta-oxidation (Pegorier et al., 1989), inhibits lipogenesis and decrease triglyceride (TG) and very-low-density lipoprotein (VLDL) secretion (Guettet et al., 1988; Bobe et al., 2003) emphasizing a potentially important role of glucagon in lipid metabolism.

## Glucagon Might Stimulate Lipolysis in Adipose Tissue in Rodents but Not in Humans

**FIGURE 1:**
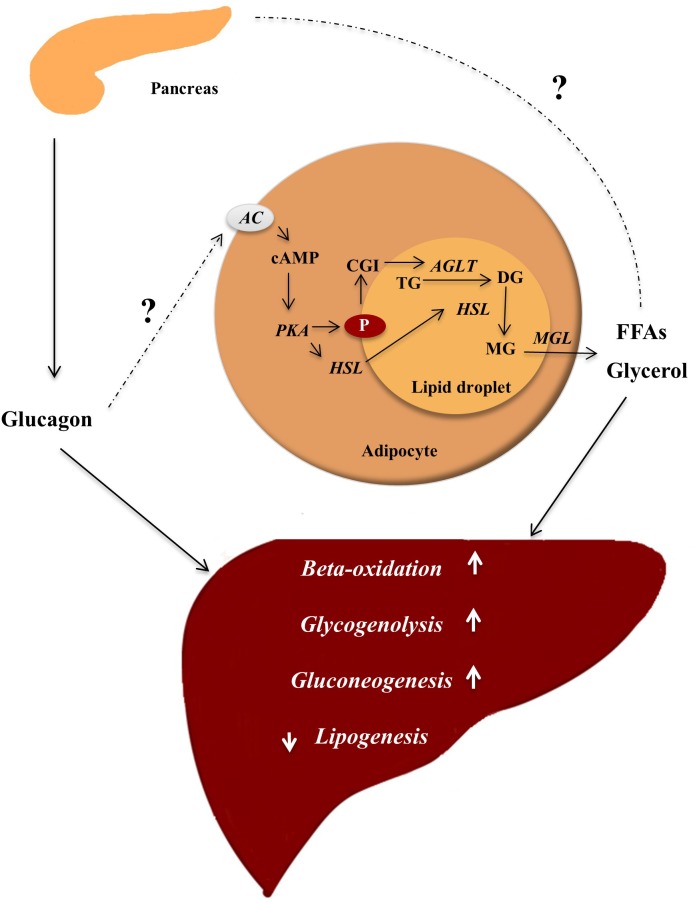
Glucagon ensures energy supply by mobilizing lipids. In the fasting state, glucagon is secreted and insulin concentrations are not sufficient to inhibit lipolysis in adipocytes, where lipids are stored in lipid droplets consisting of a core of triglycerols (TG) and sterols esters coated with perilipins (P) (proteins restricting access to the lipid core). In response to an appropriate stimuli, e.g., epinephrine and possibly glucagon, AC found in the plasma membrane of the adipocyte is activated, leading to increased intracellular concentrations of cAMP stimulating protein kinase A (PKA) activity. PKA phosphorylates (hence activates) hormone sensitive lipase (HSL) and P. The phosphorylation of P results in dissociation of the protein CGI-58. CGI-58 activates adipose triglycerol lipase (ATGL), which converts TGs to diaglycerols (DG). The phosphorylated P bind HSL and allows it to access the lipid droplet where it coverts DGs to monoglycerols (MG). The monoglycerols are hydrolyzed by monoacylglycerol lipase (MGL), yielding free fatty acids (FFAs) and glycerol, which are released to the blood. FFAs may stimulate glucagon secretion, and glucagon in turn stimulates hepatic gluconeogenesis (using FFAs and glycerol as substrates), glycogenolysis, and beta-oxidation thus providing substrates for the liver to secure sufficient energy supply to metabolically active tissue. Enzymes are written in italic and arrows indicate stimulation.

Lipolysis in adipocytes depends on activation of AC and thereby increased protein kinase A (PKA) activity. PKA phosphorylates (hence activates) perilipins (Greenberg et al., 1991) and hormone-sensitive lipase (HSL) (Stralfors et al., 1984; Garton et al., 1988; Anthonsen et al., 1998), and two additional lipases, resulting in hydrolysis of TGs and release of glycerol and free fatty acids (FFAs), e.g., palmitate (Egan et al., 1992; Lass et al., 2006; Granneman et al., 2009; Shen et al., 2009; Wang et al., 2009; Figure 1). Circulating levels of FFAs and glycerol therefore reflect the rate of lipolysis (Schweiger et al., 2014). For glucagon to directly influence adipocyte function, its cognate receptor must be expressed. Glucagon receptor mRNA has been detected in rat adipocytes (Svoboda et al., 1994; Hansen et al., 1995), but to determine the physiological relevance of glucagon receptor mRNA expression, it is necessary to investigate whether the mRNA is actually translated into a functional receptor. Specific antibodies directed against the glucagon receptor are necessary in addressing this question, but development of specific antibodies against glucagon receptors has been challenging and the antibodies available are unspecific and therefore not suitable for receptor localization (van der Woning et al., 2016). As an example, one study reported localization of the glucagon receptor in rat adipocytes using a monoclonal antibody (Iwanij and Vincent, 1990) whereas another using autoradiography, glucagon receptors were not found to be expressed (Watanabe et al., 1998), and no studies have demonstrated presence of glucagon receptors on human adipocytes (Carranza et al., 1993). Clearly, future studies should investigate glucagon receptor expression using antibody and antibody-independent methods.

Glucagon has been reported to activate HSL (Vaughan et al., 1964; Slavin et al., 1994) and lipolysis in rat adipocytes (Vaughan and Steinberg, 1963; Rodbell and Jones, 1966; Prigge and Grande, 1971; Manganiello and Vaughan, 1972; Lefebvre et al., 1973; Livingston et al., 1974) within minutes (Honnor et al., 1985) at concentrations as low as 6 × 10^−10^ M (Lefebvre and Luyckx, 1969) and 10^−11^ M (Heckemeyer et al., 1983). Glucagon has also been shown to stimulate lipolysis in birds, rabbits (Richter et al., 1989; Wu et al., 1990), and human adipocytes *in vitro* (Perea et al., 1995) at concentrations near 10^−8^ M (Richter et al., 1989). At physiological plasma concentrations (1–40 pM), a lipolytic effect of glucagon in human adipocytes has been difficult to demonstrate (Mosinger et al., 1965; Vizek et al., 1979; Gravholt et al., 2001). One of the first human studies reporting a lipolytic effect of glucagon, demonstrated that an injection of 7.5 μg glucagon into the branchial artery resulted in a rapid increase in FFA plasma concentrations in the corresponding vein (Pozza et al., 1971) but this was not replicated in a similar study with mean increases of glucagon plasma concentrations by 237 pM in overnight fasted subjects (Pozefsky et al., 1976). An increase in FFA plasma concentrations has been demonstrated upon glucagon infusion (mean glucagon increment 209 ± 15 pM) (Schneider et al., 1981) and intravenous injection of glucagon [reaching plasma concentrations of >1,000 pM (Schade and Eaton, 1975)]. Since supra-physiological glucagon concentrations were applied, these studies may lack specificity because of interaction of glucagon with other related G protein-coupled receptors (e.g., the glucagon-like peptide 1 (GLP-1) receptor) (Hjorth et al., 1994). Pharmacological concentrations of glucagon also stimulate secretion of catecholamines and growth hormone, both of which have powerful lipolytic effects (Mitchell et al., 1969; Stallknecht et al., 1995), possibly as part of a generalized sympathetic nervous system discharge (Paschoalini and Migliorini, 1990). Glucagon was not found to have any lipolytic effects in clinical studies using glucagon concentrations ranging from 19 to 64 pM (Wu et al., 1990; Jensen et al., 1991; Gravholt et al., 2001; Xiao et al., 2011). In some clinical studies investigating the lipolytic effect of supra-physiological glucagon concentrations, the lipolytic effect of glucagon could be abolished by insulin (Samols et al., 1965; Goldfine et al., 1972; Liljenquist et al., 1974; Schade and Eaton, 1975; Schneider et al., 1981), and in rat adipocytes insulin is a potent inhibitor of lipolysis (Rodbell and Jones, 1966; Lefebvre and Luyckx, 1969; Prigge and Grande, 1971; Liljenquist et al., 1974; Gerich et al., 1976). A lipolytic effect of glucagon, if any, on human adipocytes may therefore only be physiologically relevant when insulin secretion is low. Supporting this, a 2-h infusion of 1 ng/kg × min glucagon (presumably resulting in physiologically relevant elevations) and somatostatin in insulin-deficient diabetic subjects caused a two to three-fold increase in FFA and glycerol plasma concentrations, compared to infusion of somatostatin alone. However, when insulin, somatostatin, and glucagon were infused together, glucagon had no lipolytic effect (Gerich et al., 1976). Furthermore, infusion with saline only gave the same increase in FFA as compared to glucagon infusion. In another study glucagon was infused at 1.2 ng/kg × min (high but also relevant) together with somatostatin for 2 h, but there was no lipolytic effect of glucagon at insulin concentrations of 38 pM (Jensen et al., 1991). In contrast, a 2-h glucagon infusion at 1.3 ng/kg × min, during a mean insulin plasma concentration of 65 pM, increased the rate of appearance of labeled FFA and glycerol by 40 and 36%, respectively (Carlson et al., 1993). As glucagon receptors are expressed on beta cells (Adriaenssens et al., 2016; Svendsen et al., 2018) and may stimulate insulin secretion through both GLP-1 and glucagon receptors (Svendsen et al., 2018) it may be speculated that intraislet regulation of insulin through glucagon may contribute to its effect on lipid metabolism.

It is important to note that FFA and glycerol in plasma are not only determined by release from adipocytes, but also by rate of uptake and re-esterification in other tissues. A lack of effect of glucagon on the free plasma pool of FFA and glycerol, does therefore not rule out that glucagon has a direct effect on lipid metabolism in adipocytes and hepatocytes (Figure 1).

## Glucagon Stimulates Hepatic Beta-Oxidation and Inhibits Lipogenesis

**FIGURE 2:**
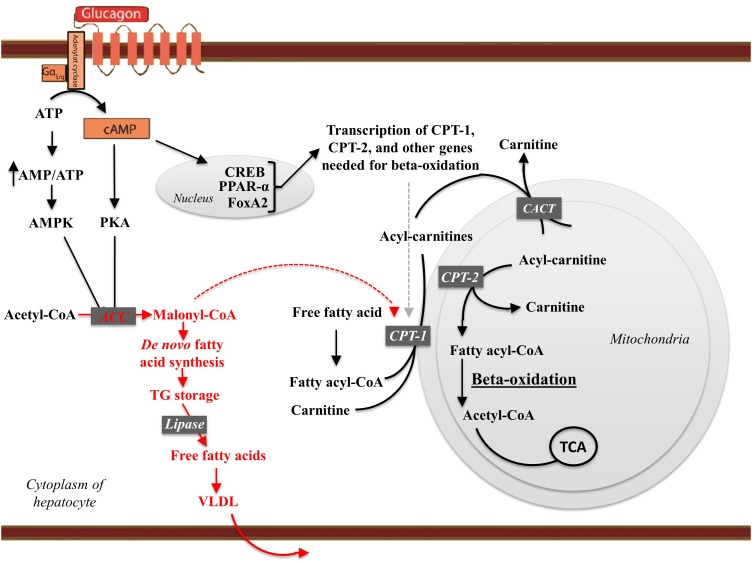
The effects of glucagon receptor signaling on hepatic lipid metabolism. Glucagon activates its cognate receptor, a seven transmembrane receptor coupled to a Gs protein, resulting in AC activity and cAMP production. The increase in intracellular cAMP activates protein kinase A (PKA), which phosphorylates (hence inactivates) acetyl-CoA carboxylase (ACC). Glucagon thus inhibit malonyl-CoA formation and the subsequent *de novo* fatty acid synthesis. When formed, the fatty acids are, after re-esterification, stored as trigycerides in and released from the hepatocytes in the form of very-low density lipoprotein (VLDL). Thus, glucagon leads the free fatty acids toward beta-oxidation and decreases *de novo* fatty acid synthesis and VLDL release. cAMP accumulation in hepatocytes activates the cAMP responsible binding element (CREB) protein, which induces the transcription of carnitine acyl transferase-1 (CPT-1), and other genes needed for beta-oxidation. CPT-1 catalyzes the attachment of carnitine to fatty acyl-CoA, forming acyl-carnitine. The acyl-carnitines transverse the mitochondrial membrane mediated via the carnitine-acylcarnitine translocase (CACT). Once in the mitochondrial matrix, carnitine acyl transferase-2 (CPT-2) is responsible for transferring the acyl-group from the acyl-carnitine back to CoA. Carnitine leaves the mitochondria matrix through the carnitine-acylcarnitine translocase. During beta-oxidation, the fatty acid chains are degraded into acetate. Acetate reacts with CoA to yield acetyl-CoA, which reacts with oxaloacetate to form citrate that inhibits glycolysis through inhibition of pyruvate dehydrogenase and phosphofructokinase-1. Finally, citrate enters the citric acid cycle (TCA). Thus, glucagon increases fatty acid catabolism, inhibits glycolysis, and fuels the TCA cycle. By increasing AC activity glucagon increase the AMP/ATP ratio sufficient to activate AMP-activated kinase (AMPK), which phosphorylates ACC, leading to transcriptional activation of peroxisome proliferator-activated receptor-α (PPARα). PPARα stimulates the transcription of genes involved in beta-oxidation including CPT-1, CPT-2, and acetyl-CoA oxidase. Glucagon stimulates FoxA2 activity, which induces transcription of genes such as CPT-1, very-, and medium- long-chain acyl-CoA dehydrogenase. Enzymes and pathways inhibited by glucagon are shown in red, while enzymes and pathways stimulated by glucagon are shown in black.

In hepatocytes, glucagon action increases the transcription factor cAMP responsive element binding (CREB) protein, which induces the transcription of carnitine acyl transferase 1 (CPT-1) (Longuet et al., 2008). CPT-1 enables catabolism of long-chain fatty acids by converting fatty acids to acyl-carnitines, which are transported into the mitochondria and subjected to beta-oxidation (Kim et al., 2000; Stephens et al., 2007). During beta-oxidation the fatty acids are degraded into acetate, which ultimately enters the citric acid cycle (DiMarco and Hoppel, 1975). Furthermore, through PKA-dependent phosphorylation, glucagon receptor signaling inactivates acetyl-CoA carboxylase, the enzyme catalyzing the formation of malonyl-CoA. Malonyl-CoA is the first intermediate in fatty acid synthesis and inhibits CPT-1 (i.e., inhibits beta-oxidation). By inhibiting the formation of malonyl-CoA, glucagon diverts FFAs to beta-oxidation rather than re-esterification into TGs (Figure 2). Periportal and perivenous hepatocytes receive different concentrations of substrates and oxygen and as a consequence periportal hepatocytes primarily mediate oxidative processes, including beta-oxidation, whereas perivenous hepatocytes preferentially mediate glucose uptake and lipogenesis (Jungermann, 1988; Guzman and Castro, 1989).

In hepatocytes, glucagon may bring about an energy-depleted state (increasing the AMP/ATP ratio) sufficient to activate AMP-activated kinase (Berglund et al., 2009), which phosphorylates acetyl-CoA carboxylase (Peng et al., 2012) and p38 mitogen-activated protein kinase, leading to transcriptional activation of peroxisome proliferator-activated receptor-α (PPARα) (Longuet et al., 2008). PPARα stimulates the transcription of genes involved in beta-oxidation including CPT-1, CPT-2, and acetyl-CoA oxidase (Patsouris et al., 2006), and the transcription of fibroblast growth factor 21, which is produced in the liver in response to glucagon (Xu et al., 2009; Cyphert et al., 2014). Glucagon also stimulates forkhead transcription factor A2 activity (FoxA2), which induces transcription of genes involved in beta-oxidation, such as CPT-1, very-, and medium- long-chain acyl-CoA dehydrogenase (Wolfrum and Stoffel, 2006; von Meyenn et al., 2013). Subsequent to activating its receptors on hepatocytes, insulin suppresses most of these pathways, and the metabolic state in the hepatocytes may therefore be determined by the insulin-glucagon ratio, rather than by the hormone concentrations *per se* (Parrilla et al., 1974). Insulin inhibits lipolysis in adipocytes and by reducing the amount of substrate (FFA and glycerol) reaching the liver may reduce (Perry et al., 2015) hepatic gluconeogenesis.

To investigate the physiological effects of glucagon in lipid metabolism, several studies have relied on glucagon receptor knockout (*Gcgr*^−/−^) mice or animals treated with GRA. In the livers of *Gcgr*^−/−^ mice there is an increase in glycolysis and a decrease in gluconeogenesis and citric acid cycle activity, which results in decreased acetyl-CoA oxidation and acetyl-CoA accumulation. The accumulation of acetyl-CoA in the cytosol of hepatocytes results in increased lipogenesis. Supporting this, genes involved in lipogenesis, e.g., ATP citrate lyase and fatty acid synthase, were found to be upregulated in livers of *Gcgr*^−/−^ mice at both the mRNA and protein level (Longuet et al., 2008; Yang et al., 2011), while CPT-1 and -2 levels, and other enzymes necessary for beta-oxidation, were downregulated (Yang et al., 2011). Hepatic beta-oxidation is essential for the production of both glucose and ketones since it provides the substrates acetyl-CoA and acetate and mitochondrial energy supply (ATP/NADH) needed for gluconeogenesis (Staehr et al., 2003). The hepatic gene expression profile changes markedly in response to fasting, and major differences have been reported in expression levels of genes involved in lipid metabolism between the fed and fasted state (Longuet et al., 2008; Zhang et al., 2011). Following a prolonged fast (16 h), wild-type mice had an increased hepatic expression of genes involved in beta-oxidation, such as CPT-1, CPT-2, and acyl-CoA dehydrogenase, but this was not observed in *Gcgr*^−/−^ mice, which displayed an impaired beta-oxidation in both the fasted and fed state (Longuet et al., 2008) and *Gcgr*^−/−^ mice failed to change the hepatic energy state in response to fasting (Berglund et al., 2009). Furthermore, *Gcgr*^−/−^ mice showed increased hepatic TG secretion and increased plasma concentrations of TG and FFA after a 16 h fasting period, but not after 5 h of fasting (Longuet et al., 2008). Others (Gelling et al., 2003) also found similar TG and FFA plasma concentrations in *Gcgr*^−/−^ and wild-type mice after a short-term fast; they did, however, find increased plasma concentrations of LDL in *Gcgr*^−/−^ mice. Glucagon thus seems to regulate hepatic metabolism in response to fasting by stimulating glucose-producing processes, including beta-oxidation. When challenged with a high fat diet (HFD) for 8 weeks, *Gcgr*^−/−^ mice did not increase the amount of inguinal and epididymal fat, whereas the amount of both doubled in wild-type mice (Longuet et al., 2008). In line with this, others (Gelling et al., 2003) showed a decrease in white adipose tissue mass and an increase in lean body mass in *Gcgr*^−/−^ compared to wild-type mice, without changes in bodyweight, food consumption, or energy expenditure and one group (Conarello et al., 2007) found that *Gcgr*^−/−^ mice had lower amounts of white adipose tissue when fed both a HFD and a low fat diet compared to wild-type mice, and thus seemed to be resistant to diet-induced obesity. This could reflect an inability of *Gcgr*^−/−^ mice to mobilize the hepatic lipid storage; instead adipocyte lipolysis (by catecholamines) maintain the energy supply to other metabolically active tissues.

## Implications of Glucagon Receptor Signaling in the Development of Steatosis

Administration of GRAs has been associated with increased hepatic fat content (assessed as hepatic fat fraction measured by magnetic resonance imaging) and increased plasma concentrations of LDL (Guzman et al., 2017). Furthermore, subjects with endogenous glucagon deficiency (pancreatectomized subjects) (Dresler et al., 1991) and rats (Sloop et al., 2004) and diabetic (db/db) mice (Liang et al., 2004) treated with glucagon antisense oligonucleotide have increased hepatic fat. These data suggest that inhibition of glucagon receptor signaling results in hepatic lipid accumulation. In addition, *Gcgr*^−/−^ mice may be prone to steatosis when challenged with a high fat diet (HFD) for 8 weeks (Longuet et al., 2008). However, a study involving a similar HFD diet for 12 weeks and mice with the same sex, gene modification, and background (C57BL/6J), showed that *Gcgr*^−/−^ mice were protected from steatosis (Conarello et al., 2007). Of notice, C57BL/6J mice do not consistently develop steatosis upon HFD feeding (Charlton et al., 2011), and this might have influenced the results. In rats, impaired glucagon action also associates with development of hepatic steatosis (Charbonneau et al., 2005a). Interestingly, HFD feeding has been reported to decrease glucagon receptor expression at the plasma membrane of rat hepatocytes (Charbonneau et al., 2005b, 2007). These data suggest that hepatic lipid accumulation may cause impaired glucagon receptor signaling, and that this (as demonstrated using GRAs) may contribute to and accelerate hepatic lipid accumulation.

Acute administration of 30 μg/kg glucagon decreased FFA and TG plasma concentrations and reduced hepatic TG content and secretion in mice (Longuet et al., 2008). Chronic hyperglucagonemia (injection of 10 μg glucagon every 8 h for 21 days) had hypolipidemic effects in rats, evident by a 70 and 38% decrease in plasma concentrations of TGs and phospholipids, respectively (Guettet et al., 1988). Consistent with this, glucagon inhibited synthesis and secretion of TGs in cultured hepatocytes (Longuet et al., 2008), in perfused rat livers (Penhos et al., 1966; Heimberg et al., 1969), and decreased the synthesis of hepatic VLDL in rats (Eaton, 1973). In humans, hyperglucagonemia (56 ± 20 pM), during a pancreatic clamp, reduced hepatic lipoprotein particle turnover (Xiao et al., 2011), and glucagon administration increased hepatic beta-oxidation in humans (Prip-Buus et al., 1990). In diet-induced obese (DIO) mice, a once-weekly treatment with 70 nmol/kg glucagon/GLP-1 receptor co-agonist resulted in loss of fat mass, which in the same study was also found, although less pronounced in GLP-1 receptor knockout mice, and improved hepatic lipid metabolism and steatosis within 4 weeks (Day et al., 2009). Another glucagon/GLP-1 co-agonist (1.9 μmol/kg daily for 14 days) decreased acetyl-CoA and malonyl-CoA concentrations and increased CPT-1 mRNA in the livers of DIO mice, whereas a selective GLP-1 receptor agonist had no effect (Pocai et al., 2009). Both of these dual agonists reduced hepatic steatosis, increased HSL activity in adipocytes, and improved dyslipidemia in DIO mice (Day et al., 2009; Pocai et al., 2009). Supporting these data, other glucagon/GLP-1 receptor co-agonists have been reported to lower plasma concentrations of TG and cholesterol (Clemmensen et al., 2014), decrease hepatic fat content (Henderson et al., 2016), and reduce adipose mass in rodent models of T2D and obesity (Evers et al., 2017; Zhou et al., 2017). Importantly, acute administration of 25 nmol/kg glucagon/GLP-1 co-agonist decreased plasma concentration of TGs, cholesterol, and LDL in DIO mice within 1 h, whereas liraglutide (a pure GLP-1 receptor agonist) administration had no effect (More et al., 2017). In addition, hepatic synthesis of VLDL and palmitate, and fatty acid esterification decreased, while beta-oxidation and LDL receptors expression increased upon co-agonist, but not liraglutide, administration (More et al., 2017). The inhibitory effect on hepatic lipogenesis and stimulatory effect on beta-oxidation therefore seems to be mediated by glucagon receptor signaling. Several clinical studies are currently investigating the potential treatment of obesity and T2D using glucagon/GLP-1 co-agonists (Capozzi et al., 2018).

## Regulation of Glucagon Secretion by Lipids

FFAs are under certain circumstances insulin secretagogs (Boden and Carnell, 2003) but their ability to stimulate glucagon secretion remains debated (Gerich et al., 1974; Bollheimer et al., 2004; Gromada et al., 2007). Some clinical studies found a suppression of glucagon secretion at increased FFA concentrations (Madison et al., 1968; Edwards and Taylor, 1970; Luyckx and Lefebvre, 1970; Gerich et al., 1974) whereas isolated alpha cells were shown to secrete glucagon in response to FFA stimulation (Gross and Mialhe, 1986; Collins et al., 2008). In isolated rat pancreatic islets, palmitate stimulated glucagon secretion (Gremlich et al., 1997; Dumonteil et al., 2000). Others found palmitate to stimulate glucagon secretion in a glucose-dependent manner using isolated pancreatic islets; increasing at glucose concentrations of 2.8, 5.6, and 10 mM (Olofsson et al., 2004) but not at 16.7 mM (Bollheimer et al., 2004). Medium and long-chain fatty acids (>C5) have been reported to stimulate glucagon secretion by activation of FFA receptor G protein-coupled receptor 40 (GPR40) (Wang et al., 2011; Kristinsson et al., 2017) and GPR119 (Hansen et al., 2012; Li et al., 2018), both present in the pancreatic islets (Briscoe et al., 2003). FFAs may also function as metabolic substrate and stimulate alpha cell secretion through beta-oxidation (Kristinsson et al., 2017; Briant et al., 2018). FFAs decrease secretion of somatostatin (Gromada et al., 2001), and may lower the tonic inhibition of somatostatin on alpha cells (Gromada et al., 2007; Müller et al., 2017). A clinical study investigating the effects of ingestion of lipids on hormone secretion, found no change in glucagon secretion after intravenous or oral administration of a lipid emulsion (3 ml/kg) (Lindgren et al., 2011), neither did glucagon plasma concentrations change upon a 300 min lipid infusion raising FFA plasma concentrations from 0.4 to 0.8 mM (Staehr et al., 2003). No difference in glucagon secretion was observed between subjects consuming a HFD or a low-fat diet for 2 weeks (Raben et al., 2001). In contrast to this, ingestion of long–chain fatty acids (olive oil and C8 fatty acids) lead to increased plasma concentrations of glucagon 40 min after, whereas no increase was observed after ingestion of short-chain fatty acids (C4), however, glucose-dependent insulinotropic polypeptide (GIP) concentrations also increased upon ingestion of long-chain fatty acids and this may have caused an increase in glucagon secretion (Mandoe et al., 2015). Another study observed that a meal rich in mono-unsaturated fatty acids resulted in a larger glucagon response when compared to a control meal (Sloth et al., 2009). Others also observed an increase in glucagon concentrations upon fat-enriched meals (Radulescu et al., 2010; Niederwanger et al., 2014). The glucagon response observed upon a 90 min intraduodenal infusion of linoleic, oleic, and palmitic acids were significant lower than observed upon protein infusion (Ryan et al., 2013). Studies of ability of FFAs to stimulate glucagon secretion are complex, since FFAs are found in many forms and their stimulatory effect may vary (Radulescu et al., 2010) [as is the case for incretin secretion (Feltrin et al., 2004; Thomsen et al., 1999)]. Furthermore, the increased glucagon concentrations reported in some studies may result from other proglucagon products (e.g., glicentin or oxyntomodulin), since measurements of plasma glucagon concentrations have been marred with problems regarding sensitivity and specificity (Wewer Albrechtsen et al., 2016), and further studies investigating the regulation of glucagon secretion by FFAs are needed.

## Conclusion

Glucagon may, aside from its physiological actions on glucose and amino acid metabolism, also be important for lipid metabolism via effects on hepatic beta-oxidation and lipogenesis, and potentially increased lipolysis in adipocytes. A direct role of glucagon on adipocytes may be of importance in rodents, as glucagon stimulates lipolysis (Vaughan and Steinberg, 1963; Rodbell and Jones, 1966; Prigge and Grande, 1971; Manganiello and Vaughan, 1972; Lefebvre et al., 1973; Livingston et al., 1974), whereas in humans an adipocyte-dependent lipolysis of glucagon is more complex. In both rodents and humans, glucagon is a powerful regulator of hepatic lipid metabolism (Day et al., 2009; Xiao et al., 2011) as highlighted in studies using GRAs (Guzman et al., 2017). The clinical use of GRAs is further challenged by glucagon’s role in amino acid metabolism, and blocking the glucagon receptor results in hyperaminoacidemia and eventually alpha cell hyperplasia (Holst et al., 2017b). Treatment of diabetes using the current GRAs may therefore not be feasible, however, one may speculate that targeted antagonism of glucagon signaling may circumvent these unwarranted side-effects. Currently glucagon receptor agonists, combined with GLP-1 and GIP receptor agonists, are investigated as possible therapeutic agents (Gu et al., 2011; Sadry and Drucker, 2013; Sanchez-Garrido et al., 2017; Capozzi et al., 2018). In preclinical studies, these agents improve steatosis and dyslipidemia, possibly as a consequence of regulation of hepatic lipid metabolism by glucagon agonism (Day et al., 2009).

Taken together, glucagon seems to play an important physiological role in the acute regulation of lipid metabolism but clearly further studies particularly in humans are warranted.

## Author Contributions

All authors wrote and approved the final edition of the manuscript.

## Conflict of Interest Statement

The authors declare that the research was conducted in the absence of any commercial or financial relationships that could be construed as a potential conflict of interest.

## References

[B1] Adriaenssens A. E. Svendsen B. Lam B. Y. Yeo G. S. Holst J. J. Reimann F. (2016). Transcriptomic profiling of pancreatic alpha, beta and delta cell populations identifies delta cells as a principal target for ghrelin in mouse islets. *Diabetologia* 59 2156–2165. 10.1007/s00125-016-4033-1 27390011 PMC5016554

[B2] Ahren B. (2015). Glucagon-early breakthroughs and recent discoveries. *Peptides* 67 74–81. 10.1016/j.peptides.2015.03.011 25814364

[B3] Anthonsen M. W. Ronnstrand L. Wernstedt C. Degerman E. Holm C. (1998). Identification of novel phosphorylation sites in hormone-sensitive lipase that are phosphorylated in response to isoproterenol and govern activation properties in vitro. *J. Biol. Chem.* 273 215–221. 10.1074/jbc.273.1.215 9417067

[B4] Aromataris E. C. Roberts M. L. Barritt G. J. Rychkov G. Y. (2006). Glucagon activates Ca2+ and Cl- channels in rat hepatocytes. *J. Physiol.* 573(Pt 3), 611–625. 10.1113/jphysiol.2006.109819 16581855 PMC1779747

[B5] Baron A. D. Schaeffer L. Shragg P. Kolterman O. G. (1987). Role of hyperglucagonemia in maintenance of increased rates of hepatic glucose output in type II diabetics. *Diabetes* 36 274–283. 10.2337/diab.36.3.274 2879757

[B6] Berglund E. D. Lee-Young R. S. Lustig D. G. Lynes S. E. Donahue E. P. Camacho R. C. (2009). Hepatic energy state is regulated by glucagon receptor signaling in mice. *J. Clin. Invest.* 119 2412–2422. 10.1172/JCI38650 19662685 PMC2719934

[B7] Bobe G. Ametaj B. N. Young J. W. Beitz D. C. (2003). Effects of exogenous glucagon on lipids in lipoproteins and liver of lactating dairy cows. *J. Dairy Sci.* 86 2895–2903. 10.3168/jds.S0022-0302(03)73886-7 14507025

[B8] Boden G. Carnell L. H. (2003). Nutritional effects of fat on carbohydrate metabolism. *Best Pract. Res. Clin. Endocrinol. Metab.* 17 399–410. 12962693 10.1016/s1521-690x(03)00032-0

[B9] Bollheimer L. C. Landauer H. C. Troll S. Schweimer J. Wrede C. E. Scholmerich J. (2004). Stimulatory short-term effects of free fatty acids on glucagon secretion at low to normal glucose concentrations. *Metabolism* 53 1443–1448. 10.1016/j.metabol.2004.06.011 15536599

[B10] Briant L. J. B. Dodd M. S. Chibalina M. V. Rorsman N. J. G. Johnson P. R. V. Carmeliet P. (2018). CPT1a-dependent long-chain fatty acid oxidation contributes to maintaining glucagon secretion from pancreatic islets. *Cell Rep.* 23 3300–3311. 10.1016/j.celrep.2018.05.035 29898400 PMC6581793

[B11] Briscoe C. P. Tadayyon M. Andrews J. L. Benson W. G. Chambers J. K. Eilert M. M. (2003). The orphan G protein-coupled receptor GPR40 is activated by medium and long chain fatty acids. *J. Biol. Chem.* 278 11303–11311. 10.1074/jbc.M211495200 12496284

[B12] Capozzi M. E. DiMarchi R. D. Tschop M. H. Finan B. Campbell J. E. (2018). Targeting the incretin/glucagon system with triagonists to treat diabetes. *Endocr. Rev.* 39 719–738. 10.1210/er.2018-00117 29905825 PMC7263842

[B13] Carlson M. G. Snead W. L. Campbell P. J. (1993). Regulation of free fatty acid metabolism by glucagon. *J. Clin. Endocrinol. Metab.* 77 11–15. 8100827 10.1210/jcem.77.1.8100827

[B14] Carranza M. C. Simon M. A. Torres A. Romero B. Calle C. (1993). Identification of glucagon receptors in human adipocytes from a liposarcoma. *J. Endocrinol. Invest.* 16 439–442. 10.1007/BF03348878 8396602

[B15] Charbonneau A. Couturier K. Gauthier M. S. Lavoie J. M. (2005a). Evidence of hepatic glucagon resistance associated with hepatic steatosis: reversal effect of training. *Int. J. Sports Med.* 26 432–441. 16037884 10.1055/s-2004-821225

[B16] Charbonneau A. Melancon A. Lavoie C. Lavoie J. M. (2005b). Alterations in hepatic glucagon receptor density and in Gsalpha and Gialpha2 protein content with diet-induced hepatic steatosis: effects of acute exercise. *Am. J. Physiol. Endocrinol. Metab.* 289 E8–E14. 15687107 10.1152/ajpendo.00570.2004

[B17] Charbonneau A. Unson C. G. Lavoie J. M. (2007). High-fat diet-induced hepatic steatosis reduces glucagon receptor content in rat hepatocytes: potential interaction with acute exercise. *J. Physiol.* 579(Pt 1), 255–267. 10.1113/jphysiol.2006.121954 17053032 PMC2075374

[B18] Charlton M. Krishnan A. Viker K. Sanderson S. Cazanave S. McConico A. (2011). Fast food diet mouse: novel small animal model of NASH with ballooning, progressive fibrosis, and high physiological fidelity to the human condition. *Am. J. Physiol. Gastrointest. Liver Physiol.* 301 G825–G834. 10.1152/ajpgi.00145.2011 21836057 PMC3220319

[B19] Clemmensen C. Chabenne J. Finan B. Sullivan L. Fischer K. Kuchler D. (2014). GLP-1/glucagon coagonism restores leptin responsiveness in obese mice chronically maintained on an obesogenic diet. *Diabetes* 63 1422–1427. 10.2337/db13-1609 24379349

[B20] Collins S. C. Salehi A. Eliasson L. Olofsson C. S. Rorsman P. (2008). Long-term exposure of mouse pancreatic islets to oleate or palmitate results in reduced glucose-induced somatostatin and oversecretion of glucagon. *Diabetologia* 51 1689–1693. 10.1007/s00125-008-1082-0 18622593 PMC2516194

[B21] Conarello S. L. Jiang G. Mu J. Li Z. Woods J. Zycband E. (2007). Glucagon receptor knockout mice are resistant to diet-induced obesity and streptozotocin-mediated beta cell loss and hyperglycaemia. *Diabetologia* 50 142–150. 10.1007/s00125-006-0481-3 17131145

[B22] Cyphert H. A. Alonge K. M. Ippagunta S. M. Hillgartner F. B. (2014). Glucagon stimulates hepatic FGF21 secretion through a PKA- and EPAC-dependent posttranscriptional mechanism. *PLoS One* 9:e94996. 10.1371/journal.pone.0094996 24733293 PMC3986400

[B23] Day J. W. Ottaway N. Patterson J. T. Gelfanov V. Smiley D. Gidda J. (2009). A new glucagon and GLP-1 co-agonist eliminates obesity in rodents. *Nat. Chem. Biol.* 5 749–757. 10.1038/nchembio.209 19597507

[B24] Dean E. D. Li M. Prasad N. Wisniewski S. N. Von Deylen A. Spaeth J. (2017). Interrupted glucagon signaling reveals hepatic alpha-cell axis and role for l-glutamine in alpha-cell proliferation. *Cell Metab.* 25 1362–1373.e5. 10.1016/j.cmet.2017.05.011 28591638 PMC5572896

[B25] DiMarco J. P. Hoppel C. (1975). Hepatic mitochondrial function in ketogenic states. Diabetes, starvation, and after growth hormone administration. *J. Clin. Invest.* 55 1237–1244. 10.1172/JCI108042 124319 PMC301878

[B26] Dresler C. M. Fortner J. G. McDermott K. Bajorunas D. R. (1991). Metabolic consequences of (regional) total pancreatectomy. *Ann. Surg.* 214 131–140. 10.1097/00000658-199108000-00007 1867520 PMC1358512

[B27] Dumonteil E. Magnan C. Ritz-Laser B. Ktorza A. Meda P. Philippe J. (2000). Glucose regulates proinsulin and prosomatostatin but not proglucagon messenger ribonucleic acid levels in rat pancreatic islets. *Endocrinology* 141 174–180. 10.1210/endo.141.1.7230 10614637

[B28] Eaton R. P. (1973). Hypolipemic action of glucagon in experimental endogenous lipemia in the rat. *J. Lipid Res.* 14 312–318. 9704075

[B29] Edwards J. C. Taylor K. W. (1970). Fatty acids and the release of glucagon from isolated guinea-pig islets of Langerhans incubated in vitro. *Biochim. Biophys. Acta* 215 310–315. 10.1016/0304-4165(70)90029-2 4926449

[B30] Egan J. J. Greenberg A. S. Chang M. K. Wek S. A. Moos M. C. Jr. Londos C. (1992). Mechanism of hormone-stimulated lipolysis in adipocytes: translocation of hormone-sensitive lipase to the lipid storage droplet. *Proc. Natl. Acad. Sci. U.S.A.* 89 8537–8541. 10.1073/pnas.89.18.8537 1528859 PMC49955

[B31] Evers A. Haack T. Lorenz M. Bossart M. Elvert R. Henkel B. (2017). Design of novel exendin-based dual glucagon-like peptide 1 (GLP-1)/glucagon receptor agonists. *J. Med. Chem.* 60 4293–4303. 10.1021/acs.jmedchem.7b00174 28448133

[B32] Faerch K. Vistisen D. Pacini G. Torekov S. S. Johansen N. B. Witte D. R. (2016). Insulin resistance is accompanied by increased fasting glucagon and delayed glucagon suppression in individuals with normal and impaired glucose regulation. *Diabetes* 65 3473–3481. 10.2337/db16-0240 27504013

[B33] Feltrin K. L. Little T. J. Meyer J. H. Horowitz M. Smout A. J. Wishart J. (2004). Effects of intraduodenal fatty acids on appetite, antropyloroduodenal motility, and plasma CCK and GLP-1 in humans vary with their chain length. *Am. J. Physiol. Regul. Integr. Comp. Physiol.* 287 R524–R533. 10.1152/ajpregu.00039.2004 15166004

[B34] Galsgaard K. D. Winther-Sørensen M. Ørskov C. Kissow H. Poulsen S. S. Vilstrup H. (2017). Disruption of glucagon receptor signaling causes hyperaminoacidemia exposing a possible liver - alpha-cell axis. *Am. J. Physiol. Endocrinol. Metab.* 314 E93–E103. 10.1152/ajpendo.00198.2017 28978545 PMC6048389

[B35] Garton A. J. Campbell D. G. Cohen P. Yeaman S. J. (1988). Primary structure of the site on bovine hormone-sensitive lipase phosphorylated by cyclic AMP-dependent protein kinase. *FEBS Lett.* 229 68–72. 10.1016/0014-5793(88)80799-3 3345839

[B36] Gelling R. W. Du X. Q. Dichmann D. S. Romer J. Huang H. Cui L. (2003). Lower blood glucose, hyperglucagonemia, and pancreatic alpha cell hyperplasia in glucagon receptor knockout mice. *Proc. Natl. Acad. Sci. U.S.A.* 100 1438–1443. 10.1073/pnas.0237106100 12552113 PMC298791

[B37] Gerich J. E. Langlois M. Schneider V. Karam J. H. Noacco C. (1974). Effects of alternations of plasma free fatty acid levels on pancreatic glucagon secretion in man. *J. Clin. Invest.* 53 1284–1289. 10.1172/JCI107675 4825225 PMC302615

[B38] Gerich J. E. Lorenzi M. Bier D. M. Tsalikian E. Schneider V. Karam J. H. (1976). Effects of physiologic levels of glucagon and growth hormone on human carbohydrate and lipid metabolism. Studies involving administration of exogenous hormone during suppression of endogenous hormone secretion with somatostatin. *J. Clin. Invest.* 57 875–884. 10.1172/JCI108364 820717 PMC436731

[B39] Goldfine I. D. Cerasi E. Luft R. (1972). Glucagon stimulation of insulin release in man: inhibition during hypoglycemia. *J. Clin. Endocrinol. Metab.* 35 312–315. 10.1210/jcem-35-2-312 5072359

[B40] Granneman J. G. Moore H. P. Krishnamoorthy R. Rathod M. (2009). Perilipin controls lipolysis by regulating the interactions of AB-hydrolase containing 5 (Abhd5) and adipose triglyceride lipase (Atgl). *J. Biol. Chem.* 284 34538–34544. 10.1074/jbc.M109.068478 19850935 PMC2787315

[B41] Gravholt C. H. Moller N. Jensen M. D. Christiansen J. S. Schmitz O. (2001). Physiological levels of glucagon do not influence lipolysis in abdominal adipose tissue as assessed by microdialysis. *J. Clin. Endocrinol. Metab.* 86 2085–2089. 10.1210/jc.86.5.2085 11344211

[B42] Greenberg A. S. Egan J. J. Wek S. A. Garty N. B. Blanchette-Mackie E. J. Londos C. (1991). Perilipin, a major hormonally regulated adipocyte-specific phosphoprotein associated with the periphery of lipid storage droplets. *J. Biol. Chem.* 266 11341–11346. 2040638

[B43] Gremlich S. Bonny C. Waeber G. Thorens B. (1997). Fatty acids decrease IDX-1 expression in rat pancreatic islets and reduce GLUT2, glucokinase, insulin, and somatostatin levels. *J. Biol. Chem.* 272 30261–30269. 10.1074/jbc.272.48.30261 9374511

[B44] Gromada J. Franklin I. Wollheim C. B. (2007). Alpha-cells of the endocrine pancreas: 35 years of research but the enigma remains. *Endocr. Rev.* 28 84–116. 10.1210/er.2006-0007 17261637

[B45] Gromada J. Hoy M. Buschard K. Salehi A. Rorsman P. (2001). Somatostatin inhibits exocytosis in rat pancreatic alpha-cells by G(i2)-dependent activation of calcineurin and depriming of secretory granules. *J. Physiol.* 535(Pt 2), 519–532. 11533141 10.1111/j.1469-7793.2001.00519.x PMC2278803

[B46] Gross R. Mialhe P. (1986). Free fatty acids and pancreatic function in the duck. *Acta Endocrinol.* 112 100–104. 10.1530/acta.0.1120100 2872764

[B47] Gu W. Lloyd D. J. Chinookswong N. Komorowski R. Sivits G. Jr. Graham M. (2011). Pharmacological targeting of glucagon and glucagon-like peptide 1 receptors has different effects on energy state and glucose homeostasis in diet-induced obese mice. *J. Pharmacol. Exp. Ther.* 338 70–81. 10.1124/jpet.111.179986 21471191

[B48] Guettet C. Mathe D. Riottot M. Lutton C. (1988). Effects of chronic glucagon administration on cholesterol and bile acid metabolism. *Biochim. Biophys. Acta* 963 215–223. 10.1016/0005-2760(88)90283-4 3058212

[B49] Guzman C. B. Zhang X. M. Liu R. Regev A. Shankar S. Garhyan P. (2017). Treatment with LY2409021, a glucagon receptor antagonist, increases liver fat in patients with type 2 diabetes. *Diabetes Obes. Metab.* 19 1521–1528. 10.1111/dom.12958 28371155

[B50] Guzman M. Castro J. (1989). Zonation of fatty acid metabolism in rat liver. *Biochem. J.* 264 107–113. 10.1042/bj2640107 2574974 PMC1133553

[B51] Hansen H. S. Rosenkilde M. M. Holst J. J. Schwartz T. W. (2012). GPR119 as a fat sensor. *Trends Pharmacol. Sci.* 33 374–381. 10.1016/j.tips.2012.03.014 22560300

[B52] Hansen L. H. Abrahamsen N. Nishimura E. (1995). Glucagon receptor mRNA distribution in rat tissues. *Peptides* 16 1163–1166. 10.1016/0196-9781(95)00078-X 8532603

[B53] Heckemeyer C. M. Barker J. Duckworth W. C. Solomon S. S. (1983). Studies of the biological effect and degradation of glucagon in the rat perifused isolated adipose cell. *Endocrinology* 113 270–276. 10.1210/endo-113-1-270 6861701

[B54] Heimberg M. Weinstein I. Kohout M. (1969). The effects of glucagon, dibutyryl cyclic adenosine 3’,5’-monophosphate, and concentration of free fatty acid on hepatic lipid metabolism. *J. Biol. Chem.* 244 5131–5139. 4310084

[B55] Henderson S. J. Konkar A. Hornigold D. C. Trevaskis J. L. Jackson R. Fritsch Fredin M. (2016). Robust anti-obesity and metabolic effects of a dual GLP-1/glucagon receptor peptide agonist in rodents and non-human primates. *Diabetes Obes. Metab.* 18 1176–1190. 10.1111/dom.12735 27377054 PMC5129521

[B56] Hjorth S. A. Adelhorst K. Pedersen B. B. Kirk O. Schwartz T. W. (1994). Glucagon and glucagon-like peptide 1: selective receptor recognition via distinct peptide epitopes. *J. Biol. Chem.* 269 30121–30124. 7527026

[B57] Holst J. J. Holland W. Gromada J. Lee Y. Unger R. H. Yan H. (2017a). Insulin and glucagon: partners for life. *Endocrinology* 158 696–701. 10.1210/en.2016-1748 28323959 PMC6061217

[B58] Holst J. J. Wewer Albrechtsen N. J. Pedersen J. Knop F. K. (2017b). Glucagon and amino acids are linked in a mutual feedback cycle: the liver-alpha-cell axis. *Diabetes* 66 235–240. 10.2337/db16-0994 28108603

[B59] Honnor R. C. Dhillon G. S. Londos C. (1985). cAMP-dependent protein kinase and lipolysis in rat adipocytes. II. Definition of steady-state relationship with lipolytic and antilipolytic modulators. *J. Biol. Chem.* 260 15130–15138. 3877723

[B60] Iwanij V. Vincent A. C. (1990). Characterization of the glucagon receptor and its functional domains using monoclonal antibodies. *J. Biol. Chem.* 265 21302–21308. 2174441

[B61] Jelinek L. J. Lok S. Rosenberg G. B. Smith R. A. Grant F. J. Biggs S. (1993). Expression cloning and signaling properties of the rat glucagon receptor. *Science* 259 1614–1616. 10.1126/science.8384375 8384375

[B62] Jensen M. D. Heiling V. J. Miles J. M. (1991). Effects of glucagon on free fatty acid metabolism in humans. *J. Clin. Endocrinol. Metab.* 72 308–315. 10.1210/jcem-72-2-308 1991802

[B63] Jiang G. Zhang B. B. (2003). Glucagon and regulation of glucose metabolism. *Am. J. Physiol. Endocrinol. Metab.* 284 E671–E678. 10.1152/ajpendo.00492.2002 12626323

[B64] Jungermann K. (1988). Metabolic zonation of liver parenchyma. *Semin. Liver Dis.* 8 329–341. 10.1055/s-2008-1040554 3062788

[B65] Kazda C. M. Ding Y. Kelly R. P. Garhyan P. Shi C. Lim C. N. (2016). Evaluation of efficacy and safety of the glucagon receptor antagonist LY2409021 in patients with type 2 diabetes: 12- and 24-week phase 2 studies. *Diabetes Care* 39 1241–1249. 10.2337/dc15-1643 26681715

[B66] Kazierad D. J. Bergman A. Tan B. Erion D. M. Somayaji V. Lee D. S. (2016). Effects of multiple ascending doses of the glucagon receptor antagonist PF-06291874 in patients with type 2 diabetes mellitus. *Diabetes Obes. Metab.* 18 795–802. 10.1111/dom.12672 27059951

[B67] Kazierad D. J. Chidsey K. Somayaji V. R. Bergman A. J. Calle R. A. (2018). Efficacy and safety of the glucagon receptor antagonist PF-06291874: a 12-week, randomized, dose-response study in patients with type 2 diabetes mellitus on background metformin therapy. *Diabetes Obes. Metab.* 20 2608–2616. 10.1111/dom.13440 29923286

[B68] Kim J. Okamoto H. Huang Z. Anguiano G. Chen S. Liu Q. (2017). Amino acid transporter Slc38a5 controls glucagon receptor inhibition-induced pancreatic alpha-cell hyperplasia in mice. *Cell Metab.* 25 1348–1361.e8. 10.1016/j.cmet.2017.05.006 28591637 PMC8206958

[B69] Kim J. Y. Hickner R. C. Cortright R. L. Dohm G. L. Houmard J. A. (2000). Lipid oxidation is reduced in obese human skeletal muscle. *Am. J. Physiol. Endocrinol. Metab.* 279 E1039–E1044. 10.1152/ajpendo.2000.279.5.E1039 11052958

[B70] Kristinsson H. Sargsyan E. Manell H. Smith D. M. Gopel S. O. Bergsten P. (2017). Basal hypersecretion of glucagon and insulin from palmitate-exposed human islets depends on FFAR1 but not decreased somatostatin secretion. *Sci. Rep.* 7:4657. 10.1038/s41598-017-04730-5 28680093 PMC5498543

[B71] Lass A. Zimmermann R. Haemmerle G. Riederer M. Schoiswohl G. Schweiger M. (2006). Adipose triglyceride lipase-mediated lipolysis of cellular fat stores is activated by CGI-58 and defective in Chanarin-Dorfman syndrome. *Cell Metab.* 3 309–319. 10.1016/j.cmet.2006.03.005 16679289

[B72] Lefebvre P. Luyckx A. Bacq Z. M. (1973). Effects of denervation on the metabolism and the response to glucagon of white adipose tissue of rats. *Horm. Metab. Res.* 5 245–250. 10.1055/s-0028-1093959 4731272

[B73] Lefebvre P. J. Luyckx A. S. (1969). Effect of insulin on glucagon enhanced lipolysis in vitro. *Diabetologia* 5 195–197. 10.1007/BF01213680 5373820

[B74] Li N. X. Brown S. Kowalski T. Wu M. Yang L. Dai G. (2018). GPR119 agonism increases glucagon secretion during insulin-induced hypoglycemia. *Diabetes* 67 1401–1413. 10.2337/db18-0031 29669745 PMC6014553

[B75] Liang Y. Osborne M. C. Monia B. P. Bhanot S. Gaarde W. A. Reed C. (2004). Reduction in glucagon receptor expression by an antisense oligonucleotide ameliorates diabetic syndrome in db/db mice. *Diabetes* 53 410–417. 10.2337/diabetes.53.2.410 14747292

[B76] Liljenquist J. E. Bomboy J. D. Lewis S. B. Sinclair-Smith B. C. Felts P. W. Lacy W. W. (1974). Effects of glucagon on lipolysis and ketogenesis in normal and diabetic men. *J. Clin. Invest.* 53 190–197. 10.1172/JCI107537 4808635 PMC301453

[B77] Lindgren O. Carr R. D. Deacon C. F. Holst J. J. Pacini G. Mari A. (2011). Incretin hormone and insulin responses to oral versus intravenous lipid administration in humans. *J. Clin. Endocrinol. Metab.* 96 2519–2524. 10.1210/jc.2011-0266 21593115

[B78] Livingston J. N. Cuatrecasas P. Lockwood D. H. (1974). Studies of glucagon resistance in large rat adipocytes: 125I-labeled glucagon binding and lipolytic capacity. *J. Lipid Res.* 15 26–32. 4359539

[B79] Longuet C. Sinclair E. M. Maida A. Baggio L. L. Maziarz M. Charron M. J. (2008). The glucagon receptor is required for the adaptive metabolic response to fasting. *Cell Metab.* 8 359–371. 10.1016/j.cmet.2008.09.008 19046568 PMC2593715

[B80] Luyckx A. S. Lefebvre P. J. (1970). Arguments for a regulation of pancreatic glucagon secretion by circulating plasma free fatty acids. *Proc. Soc. Exp. Biol. Med.* 133 524–528. 10.3181/00379727-133-34511 4905571

[B81] Madison L. L. Seyffert W. A. Jr. Unger R. H. Barker B. (1968). Effect on plasma free fatty acids on plasma glucagon and serum insulin concentrations. *Metabolism* 17 301–304. 10.1016/0026-0495(68)90097-8 5646200

[B82] Mandoe M. J. Hansen K. B. Hartmann B. Rehfeld J. F. Holst J. J. Hansen H. S. (2015). The 2-monoacylglycerol moiety of dietary fat appears to be responsible for the fat-induced release of GLP-1 in humans. *Am. J. Clin. Nutr.* 102 548–555. 10.3945/ajcn.115.106799 26178726

[B83] Manganiello V. Vaughan M. (1972). Selective loss of adipose cell responsiveness to glucagon with growth in the rat. *J. Lipid Res.* 13 12–16. 4333820

[B84] Mitchell M. L. Byrne M. J. Silver J. (1969). Growth-hormone release by glucagon. *Lancet* 1 289–290. 10.1016/S0140-6736(69)91041-1 4178983

[B85] More V. R. Lao J. McLaren D. G. Cumiskey A. M. Murphy B. A. Chen Y. (2017). Glucagon like receptor 1/ glucagon dual agonist acutely enhanced hepatic lipid clearance and suppressed de novo lipogenesis in mice. *PLoS One* 12:e0186586. 10.1371/journal.pone.0186586 29065174 PMC5655430

[B86] Mosinger B. Kuhn E. Kujalová V. (1965). Action of adipokinetic hormones on human adipose tissue in vitro. *J. Lab. Clin. Med.* 66 380–389. 4284421

[B87] Müller T. D. Finan B. Clemmensen C. DiMarchi R. D. Tschöp M. H. (2017). The new biology and pharmacology of glucagon. *Physiol. Rev.* 97 721–766. 10.1152/physrev.00025.2016 28275047

[B88] Niederwanger A. Ciardi C. Tatarczyk T. Khan M. I. Hermann M. Mittermair C. (2014). Postprandial lipemia induces pancreatic alpha cell dysfunction characteristic of type 2 diabetes: studies in healthy subjects, mouse pancreatic islets, and cultured pancreatic alpha cells. *Am. J. Clin. Nutr.* 100 1222–1231. 10.3945/ajcn.114.092023 25332320

[B89] Olofsson C. S. Salehi A. Gopel S. O. Holm C. Rorsman P. (2004). Palmitate stimulation of glucagon secretion in mouse pancreatic alpha-cells results from activation of L-type calcium channels and elevation of cytoplasmic calcium. *Diabetes* 53 2836–2843. 10.2337/diabetes.53.11.2836 15504963

[B90] Parrilla R. Goodman M. N. Toews C. J. (1974). Effect of glucagon: insulin ratios on hepatic metabolism. *Diabetes* 23 725–731. 10.2337/diab.23.9.725 4413056

[B91] Paschoalini M. A. Migliorini R. H. (1990). Participation of the CNS in the control of FFA mobilization during fasting in rabbits. *Physiol. Behav.* 47 461–465. 10.1016/0031-9384(90)90109-H 2193310

[B92] Patsouris D. Reddy J. K. Muller M. Kersten S. (2006). Peroxisome proliferator-activated receptor alpha mediates the effects of high-fat diet on hepatic gene expression. *Endocrinology* 147 1508–1516. 10.1210/en.2005-1132 16357043

[B93] Pegorier J. P. Garcia-Garcia M. V. Prip-Buus C. Duee P. H. Kohl C. Girard J. (1989). Induction of ketogenesis and fatty acid oxidation by glucagon and cyclic AMP in cultured hepatocytes from rabbit fetuses. Evidence for a decreased sensitivity of carnitine palmitoyltransferase I to malonyl-CoA inhibition after glucagon or cyclic AMP treatment. *Biochem. J.* 264 93–100. 10.1042/bj2640093 2557835 PMC1133551

[B94] Peng I. C. Chen Z. Sun W. Li Y. S. Marin T. L. Hsu P. H. (2012). Glucagon regulates ACC activity in adipocytes through the CAMKKbeta/AMPK pathway. *Am. J. Physiol. Endocrinol. Metab.* 302 E1560–E1568. 10.1152/ajpendo.00504.2011 22454291 PMC3378158

[B95] Penhos J. C. Wu C. H. Daunas J. Reitman M. Levine R. (1966). Effect of glucagon on the metabolism of lipids and on urea formation by the perfused rat liver. *Diabetes* 15 740–748. 10.2337/diab.15.10.740 5924837

[B96] Perea A. Clemente F. Martinell J. Villanueva-Penacarrillo M. L. Valverde I. (1995). Physiological effect of glucagon in human isolated adipocytes. *Horm. Metab. Res.* 27 372–375. 10.1055/s-2007-979981 7590626

[B97] Perry R. J. Camporez J. G. Kursawe R. Titchenell P. M. Zhang D. Perry C. J. (2015). Hepatic acetyl CoA links adipose tissue inflammation to hepatic insulin resistance and type 2 diabetes. *Cell* 160 745–758. 10.1016/j.cell.2015.01.012 25662011 PMC4498261

[B98] Pettus J. Reeds D. Cavaiola T. S. Boeder S. Levin M. Tobin G. (2018). Effect of a glucagon receptor antibody (REMD-477) in type 1 diabetes: a randomized controlled trial. *Diabetes Obes. Metab.* 20 1302–1305. 10.1111/dom.13202 29283470 PMC6181222

[B99] Pocai A. Carrington P. E. Adams J. R. Wright M. Eiermann G. Zhu L. (2009). Glucagon-like peptide 1/glucagon receptor dual agonism reverses obesity in mice. *Diabetes* 58 2258–2266. 10.2337/db09-0278 19602537 PMC2750209

[B100] Pozefsky T. Tancredi R. G. Moxley R. T. Dupre J. Tobin J. D. (1976). Metabolism of forearm tissues in man. Studies with glucagon. *Diabetes* 25 128–135. 10.2337/diab.25.2.128 1248674

[B101] Pozza G. Pappalettera A. Melogli O. Viberti G. Ghidoni A. (1971). Lipolytic effect of intra-arterial injection of glucagon in man. *Horm. Metab. Res.* 3 291–292. 10.1055/s-0028-1096783 5129992

[B102] Prigge W. F. Grande F. (1971). Effects of glucagon, epinephrine and insulin on in vitro lipolysis of adipose tissue from mammals and birds. *Comp. Biochem. Physiol. B* 39 69–82. 10.1016/0305-0491(71)90254-9 5570026

[B103] Prip-Buus C. Pegorier J. P. Duee P. H. Kohl C. Girard J. (1990). Evidence that the sensitivity of carnitine palmitoyltransferase I to inhibition by malonyl-CoA is an important site of regulation of hepatic fatty acid oxidation in the fetal and newborn rabbit. Perinatal development and effects of pancreatic hormones in cultured rabbit hepatocytes. *Biochem. J.* 269 409–415. 10.1042/bj2690409 2167069 PMC1131592

[B104] Raben A. Holst J. J. Madsen J. Astrup A. (2001). Diurnal metabolic profiles after 14 d of an ad libitum high-starch, high-sucrose, or high-fat diet in normal-weight never-obese and postobese women. *Am. J. Clin. Nutr.* 73 177–189. 10.1093/ajcn/73.2.177 11157312

[B105] Radulescu A. Gannon M. C. Nuttall F. Q. (2010). The effect on glucagon, glucagon-like peptide-1, total and acyl-ghrelin of dietary fats ingested with and without potato. *J. Clin. Endocrinol. Metab.* 95 3385–3391. 10.1210/jc.2009-2559 20444922 PMC3213865

[B106] Ramnanan C. J. Edgerton D. S. Kraft G. Cherrington A. D. (2011). Physiologic action of glucagon on liver glucose metabolism. *Diabetes Obes. Metab.* 13(Suppl. 1), 118–125. 10.1111/j.1463-1326.2011.01454.x 21824265 PMC5371022

[B107] Richter W. O. Robl H. Schwandt P. (1989). Human glucagon and vasoactive intestinal polypeptide (VIP) stimulate free fatty acid release from human adipose tissue in vitro. *Peptides* 10 333–335. 10.1016/0196-9781(89)90039-9 2755873

[B108] Rodbell M. Jones A. B. (1966). Metabolism of isolated fat cells. 3. The similar inhibitory action of phospholipase C (*Clostridium perfringens* alpha toxin) and of insulin on lipolysis stimulated by lipolytic hormones and theophylline. *J. Biol. Chem.* 241 140–142. 4285132

[B109] Rouille Y. Westermark G. Martin S. K. Steiner D. F. (1994). Proglucagon is processed to glucagon by prohormone convertase PC2 in alpha TC1-6 cells. *Proc. Natl. Acad. Sci. U.S.A.* 91 3242–3246. 10.1073/pnas.91.8.3242 8159732 PMC43552

[B110] Ryan A. T. Luscombe-Marsh N. D. Saies A. A. Little T. J. Standfield S. Horowitz M. (2013). Effects of intraduodenal lipid and protein on gut motility and hormone release, glycemia, appetite, and energy intake in lean men. *Am. J. Clin. Nutr.* 98 300–311. 10.3945/ajcn.113.061333 23803895

[B111] Sadry S. A. Drucker D. J. (2013). Emerging combinatorial hormone therapies for the treatment of obesity and T2DM. *Nat. Rev. Endocrinol.* 9 425–433. 10.1038/nrendo.2013.47 23478327

[B112] Samols E. Marri G. Marks V. (1965). Promotion of insulin secretion by glucogen. *Lancet* 2 415–416. 10.1016/S0140-6736(65)90761-0 14346763

[B113] Sanchez-Garrido M. A. Brandt S. J. Clemmensen C. Muller T. D. DiMarchi R. D. Tschop M. H. (2017). GLP-1/glucagon receptor co-agonism for treatment of obesity. *Diabetologia* 60 1851–1861. 10.1007/s00125-017-4354-8 28733905 PMC6448809

[B114] Schade D. S. Eaton R. P. (1975). Modulation of fatty acid metabolism by glucagon in man. I. Effects in normal subjects. *Diabetes* 24 502–509. 10.2337/diabetes.24.5.502 1126591

[B115] Schneider S. H. Fineberg S. E. Blackburn G. L. (1981). The acute metabolic effects of glucagon and its interactions with insulin in forearm tissue. *Diabetologia* 20 616–621. 10.1007/BF00257430 7021278

[B116] Schweiger M. Eichmann T. O. Taschler U. Zimmermann R. Zechner R. Lass A. (2014). Measurement of lipolysis. *Methods Enzymol.* 538 171–193. 10.1016/B978-0-12-800280-3.00010-4 24529439 PMC4018506

[B117] Shen W. J. Patel S. Miyoshi H. Greenberg A. S. Kraemer F. B. (2009). Functional interaction of hormone-sensitive lipase and perilipin in lipolysis. *J. Lipid Res.* 50 2306–2313. 10.1194/jlr.M900176-JLR200 19515989 PMC2759837

[B118] Slavin B. G. Ong J. M. Kern P. A. (1994). Hormonal regulation of hormone-sensitive lipase activity and mRNA levels in isolated rat adipocytes. *J. Lipid Res.* 35 1535–1541. 7806967

[B119] Sloop K. W. Cao J. X. Siesky A. M. Zhang H. Y. Bodenmiller D. M. Cox A. L. (2004). Hepatic and glucagon-like peptide-1-mediated reversal of diabetes by glucagon receptor antisense oligonucleotide inhibitors. *J. Clin. Invest.* 113 1571–1581. 10.1172/JCI20911 15173883 PMC419489

[B120] Sloth B. Due A. Larsen T. M. Holst J. J. Heding A. Astrup A. (2009). The effect of a high-MUFA, low-glycaemic index diet and a low-fat diet on appetite and glucose metabolism during a 6-month weight maintenance period. *Br. J. Nutr.* 101 1846–1858. 10.1017/S0007114508137710 19079942

[B121] Solloway M. J. Madjidi A. Gu C. Eastham-Anderson J. Clarke H. J. Kljavin N. (2015). Glucagon couples hepatic amino acid catabolism to mTOR-dependent regulation of alpha-cell mass. *Cell Rep.* 12 495–510. 10.1016/j.celrep.2015.06.034 26166562

[B122] Staehr P. Hother-Nielsen O. Landau B. R. Chandramouli V. Holst J. J. Beck-Nielsen H. (2003). Effects of free fatty acids per se on glucose production, gluconeogenesis, and glycogenolysis. *Diabetes* 52 260–267. 10.2337/diabetes.52.2.260 12540595

[B123] Stallknecht B. Simonsen L. Bulow J. Vinten J. Galbo H. (1995). Effect of training on epinephrine-stimulated lipolysis determined by microdialysis in human adipose tissue. *Am. J. Physiol.* 269(6 Pt 1), E1059–E1066. 10.1152/ajpendo.1995.269.6.E1059 8572197

[B124] Stephens F. B. Constantin-Teodosiu D. Greenhaff P. L. (2007). New insights concerning the role of carnitine in the regulation of fuel metabolism in skeletal muscle. *J. Physiol.* 581(Pt 2), 431–444. 10.1113/jphysiol.2006.125799 17331998 PMC2075186

[B125] Stralfors P. Bjorgell P. Belfrage P. (1984). Hormonal regulation of hormone-sensitive lipase in intact adipocytes: identification of phosphorylated sites and effects on the phosphorylation by lipolytic hormones and insulin. *Proc. Natl. Acad. Sci. U.S.A.* 81 3317–3321. 10.1073/pnas.81.11.3317 6374655 PMC345498

[B126] Svendsen B. Larsen O. Gabe M. B. N. Christiansen C. B. Rosenkilde M. M. Drucker D. J. (2018). Insulin secretion depends on intra-islet glucagon signaling. *Cell Rep* 25 1127–1134.e2. 10.1016/j.celrep.2018.10.018 30380405

[B127] Svoboda M. Tastenoy M. Vertongen P. Robberecht P. (1994). Relative quantitative analysis of glucagon receptor mRNA in rat tissues. *Mol. Cell. Endocrinol.* 105 131–137. 10.1016/0303-7207(94)90162-7 7859919

[B128] Thomsen C. Rasmussen O. Lousen T. Holst J. J. Fenselau S. Schrezenmeir J. (1999). Differential effects of saturated and monounsaturated fatty acids on postprandial lipemia and incretin responses in healthy subjects. *Am. J. Clin. Nutr.* 69 1135–1143. 10.1093/ajcn/69.6.1135 10357731

[B129] Unger R. H. Orci L. (1975). The essential role of glucagon in the pathogenesis of diabetes mellitus. *Lancet* 1 14–16. 10.1016/S0140-6736(75)92375-2 46337

[B130] Vajda E. G. Logan D. Lasseter K. Armas D. Plotkin D. J. Pipkin J. D. (2017). Pharmacokinetics and pharmacodynamics of single and multiple doses of the glucagon receptor antagonist LGD-6972 in healthy subjects and subjects with type 2 diabetes mellitus. *Diabetes Obes. Metab.* 19 24–32. 10.1111/dom.12752 27501510 PMC5215471

[B131] van der Woning B. De Boeck G. Blanchetot C. Bobkov V. Klarenbeek A. Saunders M. (2016). DNA immunization combined with scFv phage display identifies antagonistic GCGR specific antibodies and reveals new epitopes on the small extracellular loops. *MAbs* 8 1126–1135. 10.1080/19420862.2016.1189050 27211075 PMC4968103

[B132] Vaughan M. Berger J. E. Steinberg D. (1964). Hormone-sensitive lipase and monoglyceride lipase activities in adipose tissue. *J. Biol. Chem.* 239 401–409. 14169138

[B133] Vaughan M. Steinberg D. (1963). Effect of hormones on lipolysis and esterification of free fatty acids during incubation of adipose tissue in vitro. *J. Lipid Res.* 4 193–199. 14168151

[B134] Vizek K. Razova M. Melichar V. (1979). Lipolytic effect of TSH, glucagon and hydrocortisone on the adipose tissue of newborns and adults in vitro. *Physiol. Bohemoslov.* 28 325–331. 158772

[B135] von Meyenn F. Porstmann T. Gasser E. Selevsek N. Schmidt A. Aebersold R. (2013). Glucagon-induced acetylation of Foxa2 regulates hepatic lipid metabolism. *Cell Metab.* 17 436–447. 10.1016/j.cmet.2013.01.014 23416070

[B136] Wakelam M. J. Murphy G. J. Hruby V. J. Houslay M. D. (1986). Activation of two signal-transduction systems in hepatocytes by glucagon. *Nature* 323 68–71. 10.1038/323068a0 3018586

[B137] Wang H. Hu L. Dalen K. Dorward H. Marcinkiewicz A. Russell D. (2009). Activation of hormone-sensitive lipase requires two steps, protein phosphorylation and binding to the PAT-1 domain of lipid droplet coat proteins. *J. Biol. Chem.* 284 32116–32125. 10.1074/jbc.M109.006726 19717842 PMC2797282

[B138] Wang L. Zhao Y. Gui B. Fu R. Ma F. Yu J. (2011). Acute stimulation of glucagon secretion by linoleic acid results from GPR40 activation and [Ca2+]i increase in pancreatic islet {alpha}-cells. *J. Endocrinol.* 210 173–179. 10.1530/JOE-11-0132 21565851

[B139] Watanabe M. Hayasaki H. Tamayama T. Shimada M. (1998). Histologic distribution of insulin and glucagon receptors. *Braz. J. Med. Biol. Res.* 31 243–256. 10.1590/S0100-879X1998000200008 9686147

[B140] Wewer Albrechtsen N. J. Kuhre R. E. Windelov J. A. Orgaard A. Deacon C. F. Kissow H. (2016). Dynamics of glucagon secretion in mice and rats revealed using a validated sandwich ELISA for small sample volumes. *Am. J. Physiol. Endocrinol. Metab.* 311 E302–E309. 10.1152/ajpendo.00119.2016 27245336

[B141] Wolfrum C. Stoffel M. (2006). Coactivation of Foxa2 through Pgc-1beta promotes liver fatty acid oxidation and triglyceride/VLDL secretion. *Cell Metab.* 3 99–110. 10.1016/j.cmet.2006.01.001 16459311

[B142] Wu M. S. Jeng C. Y. Hollenbeck C. B. Chen Y. D. Jaspan J. Reaven G. M. (1990). Does glucagon increase plasma free fatty acid concentration in humans with normal glucose tolerance? *J. Clin. Endocrinol. Metab.* 70 410–416. 10.1210/jcem-70-2-410 1967614

[B143] Xiao C. Pavlic M. Szeto L. Patterson B. W. Lewis G. F. (2011). Effects of acute hyperglucagonemia on hepatic and intestinal lipoprotein production and clearance in healthy humans. *Diabetes* 60 383–390. 10.2337/db10-0763 20980459 PMC3028336

[B144] Xu J. Stanislaus S. Chinookoswong N. Lau Y. Y. Hager T. Patel J. (2009). Acute glucose-lowering and insulin-sensitizing action of FGF21 in insulin-resistant mouse models–association with liver and adipose tissue effects. *Am. J. Physiol. Endocrinol. Metab.* 297 E1105–E1114. 10.1152/ajpendo.00348.2009 19706786

[B145] Yang J. MacDougall M. L. McDowell M. T. Xi L. Wei R. Zavadoski W. J. (2011). Polyomic profiling reveals significant hepatic metabolic alterations in glucagon-receptor (GCGR) knockout mice: implications on anti-glucagon therapies for diabetes. *BMC Genom.* 12:281. 10.1186/1471-2164-12-281 21631939 PMC3130710

[B146] Zhang F. Xu X. Zhou B. He Z. Zhai Q. (2011). Gene expression profile change and associated physiological and pathological effects in mouse liver induced by fasting and refeeding. *PLoS One* 6:e27553. 10.1371/journal.pone.0027553 22096593 PMC3212576

[B147] Zhou J. Cai X. Huang X. Dai Y. Sun L. Zhang B. (2017). A novel glucagon-like peptide-1/glucagon receptor dual agonist exhibits weight-lowering and diabetes-protective effects. *Eur. J. Med. Chem.* 138 1158–1169. 10.1016/j.ejmech.2017.07.046 28772236

